# Diagnostic Accuracy of Fine-Needle Aspiration Cytology in Cervical Lymphadenopathies at a Tertiary Care Center in the Kingdom of Bahrain

**DOI:** 10.7759/cureus.62150

**Published:** 2024-06-11

**Authors:** Kawthar Qader, Maryam Qader, Aalaa Mubarak, Khadija Alaradi

**Affiliations:** 1 Otolaryngology - Head and Neck Surgery, Salmaniya Medical Complex, Manama, BHR; 2 Otolaryngology - Head and Neck Surgery, Arabian Gulf University, Manama, BHR; 3 Laboratory and Pathology, Salmaniya Medical Complex, Manama, BHR

**Keywords:** fine-needle aspiration cytology, fine-needle aspiration cytology (fnac), excisional biopsy, histological diagnosis, cervical lymphadenopathy, diagnostic test accuracy

## Abstract

Introduction

Fine-needle aspiration cytology (FNAC) has become widely used as a first-line diagnostic tool in the evaluation of cervical lymphadenopathies (LADs). However, there are conflicting reports regarding its accuracy in differentiating between malignant and benign pathologies. In this study, we aim to determine the reliability of FNAC in distinguishing between benign and malignant pathologies causing cervical LAD.

Methods

This is a cross-sectional study reviewing the electronic medical records of all patients who underwent both FNAC and excisional biopsy of cervical LADs between January 2016 and December 2023 at a tertiary care center in the Kingdom of Bahrain. A comparison was conducted between the cytopathological results obtained by FNAC and the histopathological results obtained by excisional biopsy to determine the diagnostic accuracy of FNAC.

Results

In the study period, 83 patient records were reviewed and included in the data analysis. Fine-needle aspiration cytology yielded a sensitivity of 89.3%, a specificity of 55.6%, a positive predictive value (PPV) of 72.4%, a negative predictive value (NPV) of 80.0%, and an overall accuracy of 74.7% in diagnosing cervical LADs.

Conclusion

Despite FNAC being accessible, convenient, and cost-effective, it has certain limitations that can restrict its accuracy in diagnosing lymphomas. We recommend further studies to research these limitations and the possible tools, such as ancillary testing, that may be useful in overcoming them.

## Introduction

Cervical lymphadenopathy (LAD) is a common presenting complaint seen in clinical settings. Classically, the primary method for evaluating cervical LAD has been an open-neck excisional biopsy. Despite providing an accurate histopathological diagnosis, excisional biopsy has been associated with local complications such as scarring, local wound infections, and an increased risk of tumor seeding in cases of metastatic malignancies [1].

Consequently, fine-needle aspiration cytometry (FNAC) has rapidly become the first-line diagnostic tool in the evaluation of neck masses. It has been proven to be a less invasive test with rapidly available results [2]. Many authors report that FNAC is a valuable diagnostic tool for distinguishing between benign and malignant pathologies while also providing vital staging information in patients with established malignancies [3]. However, despite many reports claiming the high diagnostic accuracy of FNAC [3-5], there have been several reports to the contrary [6-8].

Therefore, the aim of this study is to report the results of the FNAC of cervical LADs at our center and determine its reliability in differentiating malignant from benign pathologies.

## Materials and methods

Study design and setting

This is a single-center, retrospective, cross-sectional study spanning a total of eight years, from January 1, 2016 to December 31, 2023. This study was carried out at Salmaniya Medical Complex (SMC), a tertiary care center located in Manama, in the Kingdom of Bahrain. Ethical approval was obtained from the Research Committee for Government Hospitals (approval number: 32-110324).

Participants

During the study period, 1,100 patients underwent an excisional biopsy of a neck swelling or a lymph node in the head and neck region. Of these, 83 patients were identified who fulfilled the inclusion and exclusion criteria detailed below, and all 83 patients were included in the data analysis.

Inclusion and exclusion criteria

Inclusion Criteria

All patients who underwent FNAC of cervical lymph nodes were followed by a subsequent excisional biopsy of the same site from January 1, 2016, to December 31, 2023.

Exclusion Criteria

Any patient who underwent only FNAC or an excisional biopsy of the cervical LAD was excluded from the study. In addition, any insufficient or non-diagnostic result, whether from FNAC or the excisional biopsy, led to the exclusion of these cases. Finally, patients with incomplete data or whose medical records were inaccessible due to system error were not included in the study.

Data collection

The study period was selected based on accessibility in our setting, as electronic medical records were introduced in 2016. Consequently, the collection of data started from the beginning of 2016 to the end of 2023. 

**Figure 1:**
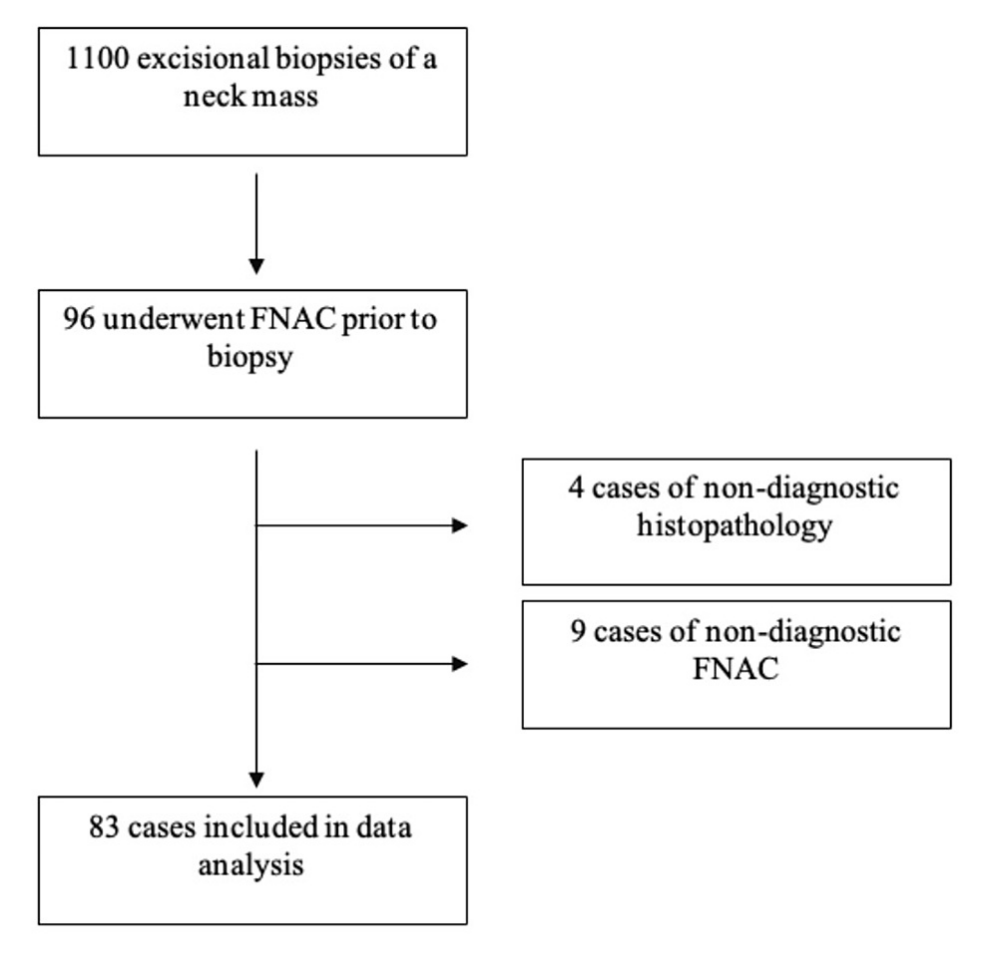
Flow diagram showcasing the selection of the study participants FNAC: fine-needle aspiration cytology

Data were extracted retrospectively from the electronic medical records to identify all patients who underwent an excisional biopsy of neck masses during the study period. This identified a total of 1,100 patients. Of these, only 96 underwent FNAC of the same cervical lymph node prior to the excisional biopsy. Of these 96 patients, nine were excluded due to the FNAC sample being insufficient or non-diagnostic, and four patients were excluded due to the biopsy sample being non-diagnostic. In the end, 83 patients were included in the analysis of the diagnostic accuracy of FNAC. This has been summarized in Figure 1.

For these 83 patients, data were collected to include the patient demographics, the location of the cervical LAD, including the side and the surgical level, the FNAC results, and the histopathological results from the excisional biopsy. All FNAC and biopsy reports were reviewed and co-signed by a senior or consultant pathologist, thereby eliminating bias or inconsistencies that may occur with pathology reports that are written by inexperienced or junior-level pathologists. 

The FNAC results of these patients were categorized as either benign or malignant and further subcategorized according to the specific pathology. This is illustrated in Table 1.

**Table 1:** Subcategories of the cytopathological diagnoses obtained by FNAC results FNAC: fine-needle aspiration cytology

Benign	Malignant
Reactive lymphoid hyperplasia	Atypical cells/suspicious for malignancy
Non-necrotizing granulomatous inflammation	Diagnostic for metastatic malignancy
Necrotizing granulomatous inflammation	Lymphoproliferative disorder/suspicious for lymphoma
	Diagnostic for lymphoma

Samples with atypia, lymphoproliferative disorders, or suspicious for malignancy were all assigned as malignant since they were all managed with a high suspicion of malignancy and investigated further. 

Histopathological diagnosis was considered the gold standard [9], and all FNAC results were compared and analyzed according to the corresponding histopathology in order to calculate the diagnostic accuracy of FNAC.

Data analysis

The data were analyzed using Microsoft Excel (Microsoft Corporation, Redmond, WA) to determine the demographics of the population and to calculate the sensitivity, specificity, positive predictive value (PPV), negative predictive value (NPV), and overall accuracy of FNAC in cervical LAD. Further analysis was done to compare the cytopathological results with the histopathological results in each of the above-mentioned cytopathological subcategories.

## Results

Demographics

A total of 83 cases were included in the data analysis. Of these, four (4.8%) cases were aged 10 years or less. A total of 18 (21.7%) cases belonged to the age group of 11-20 years. A total of 14 (16.9%) cases belonged to the age group 21-30 years, and another 14 (16.9%) cases belonged to the age group of 31-40 years. A further 13 (15.7%) cases belonged to the age group of 51-60 years. Finally, nine (10.8%) cases were aged 61 years or more. Among the 83 cases studied, 46 (55.4%) were male and 37 (44.6%) were female.

In regards to location, left-sided cervical LAD was slightly more common than the right side, with 44 (53%) cases seen on the left side and 37 (44.6%) cases on the right side. The majority of the studied cervical lymph nodes were located at surgical level three of the neck, with 38 (45.8%) cases found.

The detailed distribution of cervical LAD according to age, gender, and location is shown in Table 2.

**Table 2:** Demographical characteristics of the studied patients and location of cervical lymphadenopathy

Characteristic	Subcategory	Frequency, n (%)
Age (years)	<=10	4 (4.8%)
	11-20	18 (21.7%)
	21-30	14 (16.9%)
	31-40	14 (16.9%)
	41-50	11 (13.3%)
	51-60	13 (15.7%)
	>=61	9 (10.8%)
Gender	Male	46 (10.8%)
	Female	37 (44.6%)
Side	Right	37 (44.6%)
	Left	44 (53.0%)
	Midline	2 (2.4%)
Surgical level in the neck	1	3 (3.6%)
	2	10 (12.0%)
	3	38 (45.8%)
	4	18 (21.7%)
	5	13 (15.7%)
	6	1 (1.2%)

In order to study the frequency of different pathologies in our sample, we looked at the final histopathological diagnosis and its distribution according to age and gender. We found that two (50%) patients aged 10 years or less had cervical LAD due to Hodgkin's lymphoma (HL), and the other two (50%) were due to reactive changes. In the age group 11-20 years, nine (50%) cases of cervical LAD were due to HL, and the other nine (50%) cases were attributed to benign pathologies, most commonly reactive changes, with seven (38.9%) cases. The frequency of cervical LAD being caused by non-Hodgkin's lymphoma (NHL) was higher with increasing age, the highest being in the age group of ≥61 years with four (44.4%) cases. The risk of cervical LAD being caused by metastatic malignancy was the highest among the age groups of 41-50 years, with five (45.5%) cases. This is detailed in Table 3.

**Table 3:** Distribution of histopathological diagnoses according to age and gender

Parameter	Category	Histopathological diagnosis						Total (n)
		Benign			Malignant			
		Reactive lymphoid hyperplasia, n (%)	Non-necrotizing granulomatous inflammation, n (%)	Necrotizing granulomatous inflammation, n (%)	Metastatic malignancy, n (%)	Hodgkin's lymphoma, n (%)	Non-Hodgkin's lymphoma, n (%)	
Age (years)	≤10	2 (50.0%)	0 (0.0%)	0 (0.0%)	0 (0.0%)	2 (50.0%)	0 (0.0%)	4
	11-20	7 (38.9%)	1 (5.6%)	1 (5.6%)	0 (0.0%)	9 (50.0%)	0 (0.0%)	18
	21-30	6 (42.9%)	0 (0.0%)	1 (7.1%)	2 (14.3%)	5 (35.7%)	0 (0.0%)	14
	31-40	5 (35.7%)	0 (0.0%)	2 (14.3%)	5 (35.7%)	1 (7.1%)	1 (7.1%)	14
	41-50	1 (9.1%)	0 (0.0%)	1 (9.1%)	5 (45.5%)	1 (9.1%)	3 (27.3%)	11
	51-60	4 (30.8%)	0 (0.0%)	1 (7.7%)	4 (30.8%)	1 (7.7%)	3 (23.1%)	13
	≥61	2 (22.2%)	0 (0.0%)	2 (22.2%)	1 (11.1%)	0 (0.0%)	4 (44.4%)	9
Gender	Male	12 (26.1%)	0 (0.0%)	4 (8.7%)	9 (19.6%)	13 (28.3%)	8 (17.4%)	46
	Female	15 (40.5%)	1 (2.7%)	4 (10.8%)	8 (21.6%)	6 (16.2%)	3 (8.1%)	37

With regards to gender, the most common cause of LAD in the male population of our study was HL with 13 (28.3%) cases, followed by reactive lymphoid hyperplasia with 12 (26.1%) cases, metastatic malignancy with nine (19.6%) cases, NHL with eight (17.4%) cases, and necrotizing granulomatous inflammation with four (8.7%) cases. No cases of non-necrotizing granulomatous inflammation causing LAD in males were observed in our study.

On the other hand, the most common cause of LAD in the female population of our study was reactive changes, with 15 (40.5%) cases. This was followed by metastatic malignancy seen in eight (21.6%) cases, HL in six (16.2%) cases, necrotizing granulomatous inflammation in four (10.8%) cases, NHL seen in three (8.1%) cases, and finally non-necrotizing granulomatous inflammation with only one (2.7%) case. Overall, males were more likely to have a malignant pathology, with 30 (65.2%) out of the 46 males studied having a malignant pathology. In contrast, females were more likely to have a benign pathology, with 20 (54.1%) out of 37 females studied having a benign pathology. This is demonstrated in Table 4.

**Table 4:** Frequency of benign and malignant pathologies according to gender

Gender	Histopathological diagnosis		Total (n)
	Benign, n (%)	Malignant, n (%)	
Male	16 (34.8%)	30 (65.2%)	46
Female	20 (54.1%)	17 (45.9%)	37

Accuracy of FNAC in diagnosing malignancies

The cytopathological diagnoses obtained by FNAC were divided into benign and malignant pathologies. In our study, 25 (30.1%) cases were diagnosed as benign on FNAC, and 58 (69.9%) were diagnosed as malignant.

Out of the 25 benign cases on FNAC, the majority (19 cases) were diagnosed as reactive lymphoid hyperplasia. Granulomatous inflammation was diagnosed in six patients, with four being non-necrotizing and two being necrotizing.

On the other hand, the 58 malignant cytopathological diagnoses were further subdivided into four categories. Of these, 19 cases were considered suspicious for malignancy or had atypical cells, while 11 cases were diagnostic for a metastatic malignancy. Furthermore, 26 cases were suspicious for lymphoma or a lymphoproliferative disease, but only two cases were diagnostic for lymphoma. The distribution of the cytopathological diagnoses obtained by FNAC is shown in Table 5.

**Table 5:** Distribution of the cytopathological diagnoses based on FNAC FNAC: fine-needle aspiration cytology

Cytopathological diagnosis		Total, n (%)
Benign		25 (30.1%)
	Reactive lymphoid hyperplasia	19 (22.9%)
	Non-necrotizing granulomatous inflammation	4 (4.8%)
	Necrotizing granulomatous inflammation	2 (2.4%)
Malignant		58 (69.9%)
	Atypical cells/suspicious for malignancy	19 (22.9%)
	Lymphoproliferativesuspicious for lymphoma	26 (31.3%)
	Diagnostic for metastatic malignancy	11 (13.3%)
	Diagnostic for lymphoma	2 (2.4%)

The FNAC diagnoses were then compared to the histopathological diagnoses to determine the accuracy of FNAC in diagnosing malignancies overall. Out of the 25 patients diagnosed to have a benign pathology on FNAC, 20 (80%) were confirmed to be benign on histopathology, and five (20%) were found to be malignant. On the other hand, out of the 58 patients found to have malignant pathologies on FNAC, 42 (72.4%) were confirmed to be malignant, and 16 (27.6%) were found to be benign. This comparison is shown in Table 6.

**Table 6:** Comparison between cytopathological and histopathological diagnoses TN: true negative; FP: false positive; FN: false negative; TP: true positive

		Histopathology		Total
		Benign	Malignant	
Cytopathology	Benign	20 (80.0%) TN	5 (20.0%) FN	25
	Malignant	16 (27.6%) FP	42 (72.4%) TP	58
Total		36	47	83

The values for true positive (TP), true negative (TN), false positive (FP), and false negative (FN) were then used to calculate the diagnostic accuracy of FNAC, as shown in Table 7. In our study, FNAC yielded a sensitivity of 89.3%, a specificity of 55.6%, a PPV of 72.4%, and an NPV of 80.0%. The overall accuracy of FNAC was calculated to be 74.7%.

**Table 7:** Calculated accuracy of FNAC in diagnosing malignancies in cervical lymphadenopathies PPV: positive predictive value; NPV: negative predictive value; FNAC: fine-needle aspiration cytology

Accuracy parameter	Calculated value
Sensitivity	89.3%
Specificity	55.6%
PPV	72.4%
NPV	80.0%
Overall accuracy	74.7%

False positive and false negative results in FNAC

In our study, we had a total of 16 (27.6%) FPs, as shown in Table 6. Of these, 15 cases were initially diagnosed on FNAC as atypical cells/lymphoproliferative disorders but were found to actually be reactive on histopathology. One case was diagnosed on FNAC to be an oncocytic neoplasm but was also found to be reactive on histopathology.

Moreover, we had a total of five (20%) FN cases, as demonstrated in Table 6. All five cases were initially diagnosed as reactive lesions on FNAC, but the final histopathology determined that three cases were HL and the other two were NHL.

Accuracy of FNAC in diagnosing specific pathologies

The specific diagnoses attained by FNAC were also compared against the final diagnosis provided by histopathology. The details are shown in Table 8.

**Table 8:** Comparison between specific FNAC diagnoses and the corresponding histopathology FNAC: fine-needle aspiration cytology

			Histopathology						Total
			Benign			Malignant			
			Reactive lymphoid hyperplasia	Non-necrotizing granulomatous inflammation	Necrotizing granulomatous inflammation	Metastatic malignancy	Hodgkin's lymphoma	Non-Hodgkin's lymphoma	
FNAC	Benign	Reactive lymphoid hyperplasia	12	0	2	0	3	2	19
		Non-necrotizing granulomatous inflammation	0	1	3	0	0	0	4
		Necrotizing granulomatous inflammation	0	0	2	0	0	0	2
	Malignant	Atypical cells/suspicious for malignancy	7	0	1	7	2	2	19
		Diagnostic for metastatic malignancy	1	0	0	10	0	0	11
		Lymphoproliferative/suspicious for lymphoma	7	0	0	0	12	7	26
		Diagnostic for lymphoma	0	0	0	0	2	0	2
Total			27	1	8	17	19	11	

Benign Pathologies

A total of 19 patients were diagnosed on FNAC to have cervical LAD due to reactive changes. Of these, 12 were confirmed to be of a reactive nature on histopathology, while seven cases showed different diagnoses. Histopathology confirmed that of these seven cases initially diagnosed on FNAC as reactive changes, two cases were actually due to necrotizing granulomatous inflammation, three were cases of HL, and two were cases of NHL.

Cervical LAD due to granulomatous inflammation was less commonly seen than those caused by reactive changes. Only four cases were diagnosed on FNAC to have cervical LAD due to non-necrotizing granulomatous inflammation. Of these, only one was confirmed by histopathology, and three cases were attributed to necrotizing granulomatous inflammation (likely tuberculosis lymphadenitis).

Necrotizing granulomatous inflammation was diagnosed by FNAC in two cases, and both were confirmed by histopathology.

Malignant Pathologies

A total of 19 cases showed atypical cells or features suspicious for malignancy on FNAC. Out of these, 11 cases were confirmed by excisional biopsy to be malignant, with seven being caused by a metastatic malignancy, two by HL, and two by NHL. Additionally, out of the 19 cases found to have atypical or suspicious features on FNAC, eight cases were found to be benign on histopathology, with seven attributed to reactive changes and one case caused by necrotizing granulomatous inflammation. 

Furthermore, a total of 11 cases were diagnosed on FNAC to have metastatic malignancy, with histopathology confirming 10 of these cases. Only one of these cases was found to actually be due to reactive changes. A further 26 patients were diagnosed on FNAC to have features of an atypical lymphoproliferative disease or features suspicious of a lymphoma. Of these, 19 were confirmed to be lymphomas, with 12 being diagnosed as HL and seven being diagnosed as NHL. Finally, only two FNAC samples were diagnostic for HL, and these were both confirmed by the corresponding histopathology.

In addition, the accuracy of FNAC in the diagnosis of specific pathologies causing cervical LAD was determined using TP, TN, FP, and FN values for each pathology as detailed in Table 9.

**Table 9:** Summary of the frequency of true positive, true negative, false positive, and false negative values for FNAC in diagnosing specific conditions FNAC: fine-needle aspiration cytology; HL: Hodgkin’s lymphoma; NHL: Non-Hodgkin’s lymphoma; TN: true negative; TP: true positive; FN: false negative; FP: false positive

FNAC diagnosis		Histopathological diagnosis										
		Reactive lymphoid hyperplasia		Non-necrotizing granulomatous inflammation		Necrotizing granulomatous inflammation		Lymphoma (HL +NHL)		Metastatic malignancy		Total
		Yes	No	Yes	No	Yes	No	Yes	No	Yes	No	
Reactive lymphoid hyperplasia	Yes	12 (TP)	7 (FP)	-	-	-	-	-	-	-	-	19
	No	15 (FN)	49 (TN)	-	-	-	-	-	-	-	-	64
Non-necrotizing granulomatous inflammation	Yes	-	-	1(TP)	3(FP)	-	-	-	-	-	-	4
	No	-	-	0 (FN)	79(TN)	-	-	-	-	-	-	79
Necrotizing granulomatous inflammation	Yes	-	-	-	-	2(TP)	0(FP)	-	-	-	-	2
	No	-	-	-	-	6(FN)	75(TN)	-	-	-	-	81
Lymphoproliferative disease/suspicious or diagnostic for lymphoma	Yes	-	-	-	-	-	-	21(TP)	7(FP)	-	-	28
	No	-	-	-	-	-	-	9(FN)	44(TN)	-	-	55
Metastatic malignancy	Yes	-	-	-	-	-	-	-	-	10(TP)	1(FP)	11
	No	-	-	-	-	-	-	-	-	7(FN)	65(TN)	72
Total		27	56	1	82	8	75	30	53	17	66	-

The values of TP, TN, FP, and FN were used to calculate the diagnostic accuracy of FNAC. The sensitivity, specificity, PPV, NPV, and accuracy of FNAC in the diagnosis of LAD caused by reactive changes were 44.4%, 87.5%, 63.2%, 76.6%, and 73.5%, respectively. The sensitivity, specificity, PPV, NPV, and accuracy of FNAC for non-necrotizing granulomatous inflammation were 100%, 96.3%, 25.0%, 100%, and 96.4%, respectively. The sensitivity, specificity, PPV, NPV, and accuracy of FNAC for necrotizing granulomatous inflammation were 25.0%, 100%, 100%, 92.6%, and 92.8%, respectively. The sensitivity, specificity, PPV, NPV, and accuracy of FNAC for lymphomas were 70.0%, 86.3%, 75.0%, 83.0%, and 78.3%, respectively. The sensitivity, specificity, PPV, NPV, and accuracy of FNAC for metastatic malignancy were 58.8%, 98.5%, 90.9%, 90.3%, and 90.4%, respectively. This is outlined in Table 10.

**Table 10:** Accuracy of FNAC in diagnosing specific pathologies PPV: positive predictive value, NPV: negative predictive value; FNAC: fine-needle aspiration cytology

Parameter	Reactive changes	Non-necrotizing granulomatous inflammation	Necrotizing granulomatous inflammation	Lymphoma	Metastatic malignancy
Sensitivity	44.4%	100.0%	25.0%	70.0%	58.8%
Specificity	87.5%	96.3%	100.0%	86.3%	98.5%
PPV	63.2%	25.0%	100.0%	75.0%	90.9%
NPV	76.6%	100.0%	92.6%	83.0%	90.3%
Accuracy	73.5%	96.4%	92.8%	78.3%	90.4%

## Discussion

The American Academy of Otolaryngology-Head and Neck Surgery (AAO-HNS) recommends that in patients with a neck mass who are at an increased risk of malignancy and the diagnosis of the neck mass remains uncertain, an FNAC should be performed instead of an open biopsy in order to avoid the risk of local complications [2].

Fine-needle aspiration cytology has become widely used as an initial diagnostic test due to the early availability of results, simplicity, and lower rate of complications when compared to open biopsy [10]. Fine-needle aspiration cytology has also been found to be more cost-effective compared to open biopsy when utilized as the primary diagnostic tool in the diagnosis of LADs [11].

Cervical LADs are most commonly caused by a benign etiology, namely infections [12]. However, in our sample, when looking at the histopathological diagnoses of the population, we found that the majority of the cases studied were malignant, with 47 (56.6%) out of the 83 cases included in this study being malignant on an excisional biopsy. This could be attributed to the fact that the cases included in this study were only those who underwent both FNAC and an excisional biopsy. These were cases that displayed features highly suggestive of malignancy both clinically and on FNAC, prompting further investigation by excisional biopsy. Al Qout et al. [13] reported similar findings in their study and attributed this result to their setting, which, similar to ours, receives many cases suspicious of malignancy for the purpose of further investigation.

The accuracy of FNAC has been widely investigated, and multiple studies have been published addressing the use of FNAC in cervical LADs. A meta-analysis on the accuracy of FNAC at predicting malignancies when compared to histopathology demonstrated that FNAC has a sensitivity of 92.5%, a specificity of 97.8%, a PPV of 98.8%, an NPV of 86.7%, and an overall accuracy of 94.3% [14]. However, we found that FNAC yielded a sensitivity of 89.3%, a specificity of 55.6%, a PPV of 72.4%, and an NPV of 80.0%. The overall accuracy of FNAC at predicting malignancy was calculated to be 74.7%. Our results are more comparable to studies performed by Qasmi et al. [15] and by Hafez and Tahoun [16], which demonstrated an overall accuracy of 70% and 82.2%, respectively.

Moreover, when compared to studies in the nearby region, we found that our results for the accuracy of FNAC were significantly less than those published by Al Qout et al. [13], who conducted their study in the Kingdom of Saudi Arabia, a neighboring country. They reported a sensitivity of 93%, a specificity of 100%, a PPV of 100%, an NPV of 86.7%, and an overall accuracy of 95.3%.

The discordance in our results could be attributed to the higher number of FPs and FNs in our study. We found an FP rate of 27.6% out of the total cases identified on FNAC to be positive for malignancy. All but one of the FP cases were attributed to benign reactive lymphoid hyperplasia being misinterpreted as atypical lymphoproliferative disorders on FNAC. This is a recognized cause of FPs in FNAC [17]. The misinterpretation of reactive lymphoid cells as atypical lymphoid cells, or Hodgkin cells, is likely due to their overlapping morphological features [18]. In a similar study of 348 cases, more than half of the FP results were attributed to such misinterpretation [18]. This, along with a possible sampling error, likely accounts for the high number of FPs in our study.

On the other hand, we calculated a total of five (20%) FN cases. All five cases were initially diagnosed as reactive lesions on FNAC, but the final histopathology showed that all five were actually lymphoproliferative disorders, with three being diagnosed as HL and two as NHL. The misinterpretation of reactive lymphoid hyperplasia as atypical lymphoproliferative disorders is a recognized cause of FNs in FNAC [17]. This higher rate of FN results in the diagnosis of lymphoproliferative diseases by FNAC, which has been suggested by Chhieng et al. [6] to be a result of sampling errors, misinterpretation of lymphoid cells with similar morphological features, fibrosis of the involved lymph nodes, partial involvement of the lymph node by the disease, and obscuring reactive inflammatory cells. When comparing the accuracy of FNAC in the diagnosis of metastatic malignancy and lymphoproliferative disorders, the latter has been found to have a much lower sensitivity [19].

Classically, the diagnosis and classification of lymphoma subtypes were dependent on architectural patterns, which are only featured in histopathology obtained by biopsies [20]. This is especially important in the diagnosis of HL, as many different cell types may imitate the morphology of Reed-Sternberg cells or Hodgkin cells [20]. However, the latest World Health Organization (WHO) lymphoma classification minimizes the role of architectural patterns, which are characteristically seen in histopathology, and emphasizes individual cell morphology, immunophenotype, genetic features, and clinical information as important factors in recognizing lymphoma subtypes [21]. This significantly heightens the capability of FNAC to diagnose lymphoma, especially when supported with ancillary testing [20].

Other studies, including The Sydney System for Reporting Lymph Node Cytopathology (2020) and the more recent WHO Reporting System for Lymph Node Cytopathology (2023), both emphasize the importance of ancillary testing such as immunocytochemistry (ICC), special staining, flow cytometry, fluorescence or colorimetric in situ hybridization (FISH or CISH), and molecular procedures [3, 22]. These reporting systems describe how the use of ancillary tests is vital to achieving the “second diagnostic level,” which is described as identifying the specific subtypes of NHL and HL, providing specific etiologies in reactive lesions, and identifying the specific primary tumor in metastatic malignancies [3].

One example of such ancillary testing that may be beneficial in the diagnosis of lymphomas is the use of ICC. The role of ICC staining in the diagnosis of HL by FNAC has been studied by Zhang et al. [20] who found that satisfactory diagnostic immunostaining was achieved in 84.6% of primarily diagnosed HL and in 100% of recurrent cases of HL. This indicates the valuable role that ancillary testing, particularly ICC, may play in overcoming the limitations of FNAC and eliminating the need for an excisional biopsy.

In our study, however, ancillary testing is not routinely performed on FNAC samples. Consequently, in regard to the diagnosis of lymphomas, we found that FNAC had an overall accuracy of 78.3%. However, out of the 21 patients diagnosed correctly by FNAC to have lymphoproliferative disorders, only two cases were diagnostic for lymphoma. Of these two cases, one was confirmed as HL using ICC staining, while the other was diagnosed based on the presence of Reed-Sternberg cells. In the case of the other 19 samples, FNAC was able to correctly identify the sample as suspicious for a lymphoproliferative disorder or lymphoma but was unable to reach a specific diagnosis regarding the type or subtype of lymphoma. This would warrant an eventual excisional biopsy in order to reach the final diagnosis and proceed with management.

On the other hand, we found FNAC to be more reliable at diagnosing metastatic malignancies, with an overall accuracy of 90.4%. This is supported by several authors who report an accuracy of FNAC of up to 95%-100% [15-16, 19-23]. It has been suggested that since the diagnosis of metastatic carcinomas can be accomplished by relying only on cytomorphological features, unlike lymphoproliferative disorders, FNAC could be much more valuable in the diagnosis of metastatic malignancies than lymphomas. [19,24].

Additionally, in terms of benign lesions, FNAC had an accuracy of 73.5% in diagnosing reactive lymphoid lesions and an overall accuracy of 96.4% and 92.8% in diagnosing non-necrotizing and necrotizing granulomatous inflammation, respectively. However, the small number of patients in our study diagnosed with both necrotizing (n = 4, 4.8%) and non-necrotizing granulomatous inflammation (n = 2, 2.4%) on FNAC may not be representative of the accuracy of the FNAC in diagnosing granulomatous inflammation, and a larger sample may be required.

Furthermore, another recognized limitation of FNAC is sampling error, which may cause inadequate aspirates [25]. The Papanicolaou Society of Cytopathology considers a value of up to 10%-15% of inadequate aspirates as acceptable [25]. Possible causes of inadequate aspirates include improper handling of the aspirate, crush artifacts, and a lack of a qualified cytopathologist [13]. In our study, nine (9.4%) out of the 96 patients who were identified to have undergone both FNAC and an excisional biopsy, were excluded from the data analysis since the FNAC aspirates were inadequate. This value falls well within the acceptable range of inadequate aspirates. However, despite these cases being excluded from the analysis, it is important to acknowledge that three of these nine excluded cases were eventually diagnosed as malignancies upon excisional biopsy. This emphasizes the need to follow up on inadequate aspirates with further investigation to prevent overlooking such sinister pathologies.

Ultimately, in our study, certain limitations need to be addressed. The first limitation is that this is a single-center study. Another limitation is the relatively small number of participants included in this study. This is particularly evident in certain diagnoses, such as granulomatous disorders, which yielded a very small number of cases, and therefore the accuracy values calculated may not be representative and a larger sample is needed. Additionally, in our sample, we had to exclude four cases that underwent both FNAC and an excisional biopsy from the data analysis because the excisional biopsy sample did not yield a definitive diagnosis. Finally, ancillary testing such as ICC and molecular studies are not routinely performed in our center. Immunocytochemistry was performed on only one of our FNAC samples, thereby not allowing for the analysis of the value of immunostaining in FNAC. 

## Conclusions

Fine-needle aspiration cytology has been widely recommended as a first-line diagnostic tool in the diagnosis of cervical LAD in light of its reported high diagnostic accuracy. Based on our study, we conclude that FNAC can be an extremely reliable tool for the diagnosis of suspected metastatic malignancies. On the other hand, however, we believe that FNAC, without the aid of ancillary testing, might not be a reliable first investigative tool for the diagnosis of lymphomas, and its use may lead to a delayed diagnosis. We found this to be evident, firstly due to the inability of FNAC to establish a specific type or subtype of lymphoma, and secondly due to a higher rate of FPs and FNs. We propose that the main reasons for this could be the lack of ancillary testing at our center along with sampling error. Consequently, subsequent research should be directed at replicating this study on a larger sample size and investigating the impact of ancillary testing such as molecular studies and immunostaining on FNAC samples. This could be imperative in increasing the accuracy of FNAC in diagnosing lymphomas and avoiding unnecessary open excisional biopsies.
